# Variability of the Genes Involved in the Cellular Redox Status and Their Implication in Drug Hypersensitivity Reactions

**DOI:** 10.3390/antiox10020294

**Published:** 2021-02-15

**Authors:** Pedro Ayuso, Elena García-Martín, José A. G. Agúndez

**Affiliations:** Institute of Molecular Pathology Biomarkers, University Institute of Molecular Pathology Biomarkers, University of Extremadura, ARADyAL Instituto de Salud Carlos III, 10003 Cáceres, Spain; elenag@unex.es (E.G.-M.); jagundez@unex.es (J.A.G.A.)

**Keywords:** redox, hypersensitivity drug reaction, non-steroidal anti-inflammatory drugs, β-lactam antibiotics and SNPs

## Abstract

Adverse drug reactions are a major cause of morbidity and mortality. Of the great diversity of drugs involved in hypersensitivity drug reactions, the most frequent are non-steroidal anti-inflammatory drugs followed by β-lactam antibiotics. The redox status regulates the level of reactive oxygen and nitrogen species (RONS). RONS interplay and modulate the action of diverse biomolecules, such as inflammatory mediators and drugs. In this review, we address the role of the redox status in the initiation, as well as in the resolution of inflammatory processes involved in drug hypersensitivity reactions. We summarize the association findings between drug hypersensitivity reactions and variants in the genes that encode the enzymes related to the redox system such as enzymes related to glutathione: Glutathione S-transferase (*GSTM1, GSTP, GSTT1)* and glutathione peroxidase *(GPX1*), thioredoxin reductase (*TXNRD1* and *TXNRD2*), superoxide dismutase (*SOD1, SOD2*, and *SOD3*), catalase (*CAT*), aldo-keto reductase (*AKR*), and the peroxiredoxin system (*PRDX1, PRDX2, PRDX3, PRDX4, PRDX5, PRDX6*). Based on current evidence, the most relevant candidate redox genes related to hypersensitivity drug reactions are *GSTM1, TXNRD1, SOD1,* and *SOD2*. Increasing the understanding of pharmacogenetics in drug hypersensitivity reactions will contribute to the development of early diagnostic or prognosis tools, and will help to diminish the occurrence and/or the severity of these reactions.

## 1. Introduction

According to the World Health Organization (WHO), adverse drug reactions (ADRs) are defined as noxious and unintended reactions to a drug that is administered in standard doses by the proper route for prophylaxis, diagnosis, or treatment, or for the modification of physiological function [1]. For the last 30 years, ADRs have been the leading cause of both morbidity and mortality in the emergency departments and hence pose a significant burden on health care resources. Furthermore, ADRs represent one of the four leading causes of death in the developed world [2,3]. Approximately, 6–10% of ADRs belong to the Type B reactions, which include drug hypersensitivity reactions (DHRs). These are unpredictable, not related to the pharmacological action of the drug, and can be very severe and life-threatening [4,5]. Concerning DHRs, the most frequent drugs involved are non-steroidal anti-inflammatory drugs (NSAIDs), which account for 20–25% of patients evaluated in allergy units. These causative drugs are followed in frequency by β-lactam antibiotics [4].

From a mechanistic point of view, the European Academy of Allergy, Asthma, and Clinical Immunology (EAACI) has classified the NSAIDs hypersensitivity reactions into two main groups: Single NSAID induced hypersensitivity reactions, also known as selective reactions (SR), are specific immunological reactions, IgE or T-cell mediated. These reactions occur with a single NSAID whereas there is tolerance to other NSAIDs. The second group of NSAIDs hypersensitivity reactions involve several NSAIDs not structurally related and are mediated through a pharmacological mechanism linked to cyclooxygenase inhibition, although, the underlying mechanism is not fully understood [6,7,8,9]. The clinical manifestation may vary from mild to severe reactions such as life-threatening anaphylaxis [10]. Hence, the heterogenous symptoms observed and the severity of certain reactions hamper the diagnosis of NSAIDs hypersensitivity reactions [7].

Regarding hypersensitivity reactions triggered by β-lactam antibiotics, they are induced by specific immunological mechanisms and are mediated through immediate and non-immediate reactions [11,12]. Immediate reactions usually appear within the first hour and are mediated by specific IgE antibodies. The clinical manifestations include urticaria, angioedema, rhinitis, bronchospasm, and/or anaphylaxis [13]. These reactions constitute the most frequent DHRs to β-lactam antibiotics and the most common cause of anaphylaxis [14]. On the contrary, the non-immediate reactions constitute a group of entities that occur within a period of 24–48 h after the intake of the drug, although the time may be as short as 1 to 2 h. They are mediated through T-cells. The most common clinical entities are benign, like urticaria; nevertheless, severe reactions such as DRESS and TEN syndromes can also occur [15]. Although the pattern of β-lactam antibiotics consumption has been altered over the past years, amoxicillin is still the drug that most frequently induces DHRs [11]. The details about the interaction between β-lactam antibiotics and immune cell receptors have not yet been fully determined.

Furthermore, several factors may contribute to the occurrence of DHRs. In this regard, it has been pointed out that the reactive metabolites generated by the bioactivation of a particular drug and its binding to cellular macromolecules might be related to DHR development [16,17]. Thus, β-lactam antibiotics form adducts with high molecular weight proteins through a covalent bond generating a hapten-carrier formation for immune activation [18]. Also, it is demonstrated that sulfamethoxazole metabolites such as the nitroso sulfamethoxazole can form adducts with protein cysteine residues, and induce DHR reactions [19,20]. Furthermore, it has been documented that both a higher amount of reactive metabolite bound to cellular proteins and a higher total drug daily dose is correlated with an increased risk of DHR [21]. Supporting the concept that additional factors might contribute to DHR development, the danger hypothesis proposes that immune response to drugs is also determined by cell damage. Thus, injured cells might release alarm signals, such as reactive oxygen intermediates that might be capable of modifying the immune response. In this regard, the oxidative stress status may contribute as a key factor in the development of DHRs [16,22]. Genetic association studies have documented several findings supporting this hypothesis [23,24]. Thus, a previous report demonstrated an association between the risk of developing DILI in Spanish individuals carrying the double GSTT1-GSTM1 null genotypes [25]. Also, the haplotype conformed by the rs10735393, rs4964287, and rs4595619 single-nucleotide polymorphisms (SNPs) in the thioredoxin reductase 1 gene was found associated with a cohort of Asian patients with DILI [26]. Despite these previous findings, the role of redox status in the development of DHRs is scarcely studied.

The evidence raised from recent studies, particularly those from collaborative studies in the ARADyAL network suggests that redox status might play a key role in the development or the clinical presentation of DHRs [27]. Here, we address a perspective on the contribution of redox status to the development of drug hypersensitivity reactions. Moreover, we summarize the association findings between the variants in genes that encode the redox system enzymes and drug hypersensitivity reactions (Table 1), and we identify topics that should be further investigated.

## 2. Redox Status and Hypersensitivity Drug Reactions

Chemically, reactive oxygen species (ROS) and reactive nitrogen species (RNS) are a family of reactive species containing partially reduced oxygen and derived from oxygen or nitric oxide. These species include superoxide anion, hydroxyl radical, and peroxynitrite. These molecules are highly reactive and may modulate cellular signaling through the interaction with diverse biomolecules [41,42]. These biomolecules may suffer oxidative modifications which may undergo conformational changes or even severe denaturation. Oxidative stress is usually defined as increased production and/or decreased scavenging or metabolism of ROS [43]. The intracellular redox status determines the function of ROS and RNS. Thus, excessive production of these reactive species evokes cytotoxic effects that may lead to apoptotic cell death, whereas a lower level of these may mediate the regulation of certain cellular processes such as inflammation, protein expression, or posttranslational modifications. Moreover, ROS can play a role as second messengers in signaling pathways [41,44,45,46]. The mechanisms to protect biomolecules from oxidative stress are widely conserved among mammalians and constitute a complex composed of antioxidant enzymes, small antioxidant molecules, and molecules able to scavenge transition metal ions: Glutathione S-transferases (GSTM1, GSTP, GSTT1). These enzymes catalyze the nucleophilic attack by reduced glutathione (GSH) on nonpolar compounds that contain an electrophilic atom. Moreover, the glutathione peroxidase (GPX) family catalyzes the reduction of a variety of hydroperoxides using GSH. The thioredoxin reductase family (TXNRD1 and TXNRD2) degrades hydrogen peroxide and protects cells from cytotoxicity. The superoxide dismutase enzymes (SOD1, SOD2, and SOD3) decompose the free radical superoxide by converting it to hydrogen peroxide, which may, in turn, be destroyed by catalase (CAT). The aldo-keto reductase (AKR) family of enzymes which detoxifies reactive aldehydes to alcohols and peroxiredoxin system (PRDX1, PRDX2, PRDX3, PRDX4, PRDX5, PRDX6) have a crucial role as a scavenger of hydrogen peroxide [47,48,49,50].

### 2.1. Nonspecific Immunological Reactions

These are the most frequent type of DHRs. These reactions are also named cross-intolerant reactions and they are caused by two or more NSAIDs with chemically unrelated structures [4]. According to the EAACI-ENDA group, the clinical entities of this group are NSAIDs exacerbated respiratory disease (NERD), NSAIDs exacerbated cutaneous disease (NECD) and NSAIDs induced urticaria and/or angioedema (NIUA) [7]. NSAIDs exert their effect by blocking the COX enzyme’s action, which is responsible for the production of prostaglandins from arachidonic acid; and consequently, modifying eicosanoid inflammatory mediators’ production through the arachidonic acid pathway [51]. Moreover, arachidonic acid is also metabolized through the 5-LOX pathway into leukotrienes. Prostaglandins and leukotrienes develop a key role in the coordination of the initial events of inflammatory processes such as cytokine production, cellular proliferation, and migration [52]. NSAIDs intake brings about an imbalance in the inflammatory mediators’ production in individuals who manifest cross-intolerant reactions to these drugs by mechanisms that are not fully understood [53]. Hence, an increasing number of studies support the hypothesis that additional pathways may contribute toward the underlying mechanisms of DHRs to NSAIDs [54,55].

The redox status may determine the action of ROS and RNS over diverse biomolecules involved in cellular signaling pathways, such as the inflammatory response or asthma [56,57]. Thus, peroxynitrite has several effects on COX activity. This reactive compound provides the peroxide necessary for COX activation, while in the absence of arachidonic acid, peroxynitrite through nitration of a tyrosine residue (Tyr385) inhibit COX activity [58]. Besides, diverse polyunsaturated fatty acids are susceptible to oxidation. Thus, the peroxidation of these compounds leads to the generation of several reactive species, such as reactive aldehydes and F2-isoprostanes which are shown to induce infiltration and activation of inflammatory cells [41]. Interestingly, the aldo-ketoreductase enzyme AKR1B1 may regulate PGE2 production through PGF2α, which exerts a key role in the development of NSAIDs hypersensitivity reactions [53,59]. Furthermore, the oxidation of low-density lipoproteins can be specifically recognized by the receptors of immune cells and induce activation of the inflammatory response. On the contrary, high-density lipoproteins that have antioxidant and anti-inflammatory properties can be inhibited by these reactive species [60,61].

These reactive species may regulate the function and activity of many proteins through interaction and oxidation of their Cys, Tyr, and Met amino acids. These modifications are reversible, being TRX, and GSH/GRX the primary redox-sensitive reductases systems [62]. Certain proteins involved in proinflammatory processes are regulated by these redox modifications. Thus, AMPK protein kinase can be activated by ROS through redox modification of Cys299 and Cys304. Its activation can inhibit NF-κB signaling and inflammation that is mediated by different downstream targets [41,63]. In addition, the redox status can activate signal transduction cascades and induce changes in transcription factors, such as NF-κB which lead to the subsequent expression of inflammatory mediators, such as cytokines or interleukins IL-1, IL-6, and IL-8 [41,64]. The cytokines secretion is regulated by several signaling pathways involving the JAK-STAT pathways, and the STAT transcription factor family requires ROS species for their transcriptional activity [65].

Moreover, NF-κB can enhance the expression of antioxidant enzymes, such as SOD2, and might promote the biosynthesis of GSH. These compounds protect against excessive ROS accumulation revealing the permanent crosstalk between redox status and inflammatory processes [66,67]. Nrf2 is also a transcription factor involved in the inflammatory response which protects against airway inflammation and asthma. Its action is regulated by the modification of redox-sensitive cysteine residues [41,68]. These studies reflect the role of redox status as a key factor in the initiation as well as resolving inflammatory processes through interaction with regulator proteins and transcription factors that are sensitive to redox modifications.

**Figure 1:**
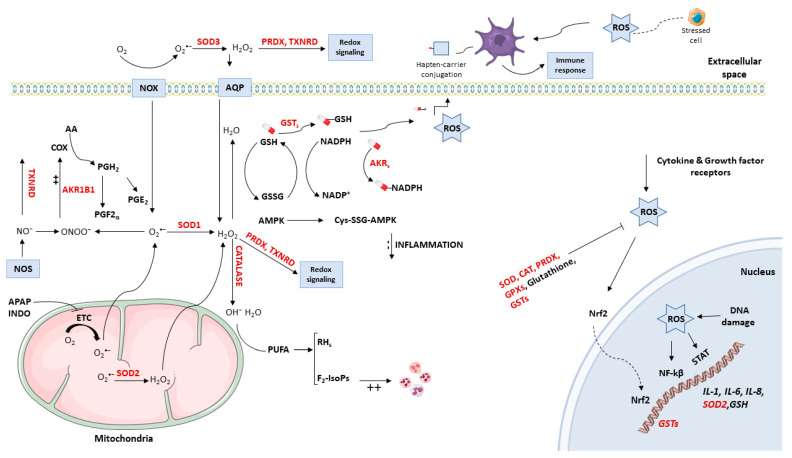
Potential role of redox regulation in the development of drug hypersensitivity reactions. The mitochondrial electron transport chain (ETC) produces superoxide (O_2_^•−^) that is converted into hydrogen peroxide (H_2_O_2_) by means of SOD2. Acetaminophen (APAP) and indomethacin (INDO) may cause mitochondrial dysfunction and alter the redox status through the inactivation of mitochondrial ETC components. The H_2_O_2_ can cross the membrane and be converted into hydroxyl radical and water by catalase. Cytosolic, polyunsaturated fatty acids (PUFA) are susceptible to oxidation and lead to the generation of reactive aldehydes (RHs) and isoprostanes. Also, the O_2_^•−^ generated by ETC can cross the mitochondrial membrane and be converted into peroxynitrite (ONOO^−^) spontaneously in the presence of nitric oxide (NO). The ONOO^−^ generated can up-regulate COX activity resulting in an increase in the production of prostanoids. Besides, the enzyme AKR1B1 may regulate PGE2 production through PGF2α reduction. The plasma membrane protein NADPH-oxidase (NOX) can generate O_2_^•−^ that is transformed into H_2_O_2_ by the extracellular SOD3. Finally, H_2_O_2_ can cross the membrane via aquaporins (AQP) or participate as a second messenger and activate effector molecules via the action of PRDX and TXNRD. These extracellular enzymes can modulate several pathophysiological processes, such as immune response and inflammation. In the cytosol, AMPK protein kinase can be activated by ROS and inhibit inflammation. The redox status can activate signal transduction cascades and induce changes in transcription factors that modulate the expression of inflammatory mediators. Drug biotransformation (either bioactivation or detoxication) may generate reactive metabolites. The most common detoxication process is carried out through conjugation with GSH. These metabolites have the potential to act as haptens and play as antigenic determinants to trigger the adaptive immune response. Moreover, the danger hypothesis proposes the necessary activation of the immune system by stressed cells, which is mediated by certain molecules that act as danger signals. Thus, molecules involved in oxidative stress response can act as potential danger signals. Besides ROS originated at the mitochondria, these species are also produced in response to cytokines and growth factor receptors. Enzymes such as CAT, PRDX, GPXs, SOD, GSTs, and GSH can reduce the cytoplasmic ROS levels. The redox status can activate signal transduction cascades and induce changes in redox-dependent transcription factors, such as NF-κB which leads to the subsequent expression of cytokines and interleukins IL-1, IL-6, and IL-8. Also, the cytokines secretion is regulated by several signaling pathways involving the JAK-STAT pathways, and the STAT transcription factor family requires ROS species for their transcriptional activity. Nrf2 is involved in the inflammatory response which protects against airway inflammation and asthma. In the nucleus, DNA damage and repair processes are a source of ROS. These redox-dependent transcription factors have cysteine residues which are also regulated by the redox status of the nucleus.

Acetaminophen, a non-NSAID drug with antipyretic and analgesic effect, has been revealed as a drug capable of blocking the electron transport from the mitochondrial respiratory chain complex I to III and consequently induces an alteration of the redox status [69]. Moreover, indomethacin may induce reactive species through the inactivation of mitochondrial aconitase, which may cause mitochondrial dysfunction and oxidative stress [70]. Whether additional NSAIDs might exert a similar action and its consequences over the redox status and the mechanisms of the DHRs to NSAIDS need to be elucidated in the foreseeable future. Figure 1 summarizes the mechanisms covered in this review.

### 2.2. Specific Immunological Reactions

Based on the timing of the symptoms, the specific immunological drug reactions may be commonly classified into two broad categories. Immediate DHRs are induced by an IgE-mediated mechanism. At an initial phase, Th2-cells promote B-cells proliferation and differentiation into plasma cells to produce drug-specific IgE. These antibodies bind to the high-affinity FcRI receptors on the surface of mast cells and basophils and their activation induces the release of preformed mediators. On the contrary nonimmediate DHRs are mediated through T-cells. Dendritic cells might process the drug antigen and subsequently stimulate naïve T cells [10,11,12]. β-lactam antibiotics are the drugs most frequently involved in DHRs induced by specific immunological reactions [4].

It has been demonstrated that β-lactam antibiotics induce immunological reactions binding irreversibly to high molecular weight proteins, thus generating a hapten-carrier formation. For protein conjugate formation, this binding occurs through the nucleophilic attack of the β-lactam ring by lysine residues and the generation of reactive intermediates. In addition, the role of side-chain structures that define different penicillin compounds as antigenic determinants is widely accepted [4,13,18]. Furthermore, multiple evidence suggests that specific immunologic DHRs might be triggered by reactive metabolites of drugs rather than by the parent drug [71]. The bioactivation of drugs may generate reactive species, and processes involved in drug biotransformation might generate reactive metabolites. These metabolites have the potential to act as haptens and play as antigenic determinants to trigger the adaptive immune response [71]. Also, the risk that a drug may cause specific immunological DHRs is linked to the amount of reactive metabolite generated [21,71]. The formation of these reactive species is often involved in toxicity. One of the major drug biotransformation processes is carried out through conjugation with GSH. Certain reactive metabolites react spontaneously with GSH, whereas others require an enzymatic action by glutathione-*S*-transferases (GST) [50,71]. Hence, the redox system might act as a source of reactive species that have the potential to bind covalently to proteins and being able to function as haptens. In addition, the hapten mechanism has also been related to the danger hypothesis mechanism [72]. Both mechanisms can explain the interaction between drugs or reactive metabolites and the immune system. They are not exclusive and may work cooperatively to explain a specific immunologic DHR. The danger hypothesis proposes the necessary activation of the immune system by damaged/stressed cells, which is mediated by certain molecules that act as danger signals. These danger signals might act as adjuvants to upregulate co-stimulatory molecules on antigen-presenting cells [17]. The nature of these danger molecules is unknown. However, they might be endogenous molecules generated because of tissue damage or cell stress caused by parent drugs or reactive metabolites. Considering the capacity of certain drugs to generate oxidative stress and its consequences, molecules involved in oxidative stress response might act as potential danger signals [22]. Therefore, both proposed mechanisms suggest a concomitant contribution of the redox status to the development of specific immunological DHRs.

The imbalanced oxidative status has been evidenced in allergy to different drugs. Hence, mononuclear cells from DHRs patients demonstrated SODs and CAT enhanced activities compared to control individuals [73]. SOD activity and expression have demonstrated being increased in patients suffering from non-immediate cutaneous reactions to drugs [74]. This crosstalk between the redox system and the adaptive immune system is also supported by the fact that it has been reported the innate immune activation following stress signaling may promote sensitization and favor Th2 responses [75].

Interestingly, an ample volume of evidence supports that enzymes involved in redox status mechanisms, such as AKR and GST enzymes can interact with the mechanism of action of drugs. In addition, these enzymes can be covalently modified and regulated by drugs and oxidative stress [76].

Elzagallaai et al. have characterized the cytotoxicity, ROS generation, carbonyl protein formation, lipid peroxidation, and GSH content in monocytes and platelets from patients with specific immune hypersensitivity to sulfonamides [16]. The sulfamethoxazole N-hydroxylamine reactive metabolite, generated through cytochrome P450 oxidation of the parental drug, can oxidize reactive cysteine thiols generating reactive oxygen species that cause cytotoxicity. In this context, hypersensitivity patients demonstrated elevated levels of ROS, significantly higher amounts of lipid peroxidation, and protein carbonylation. Consistently, these patients showed significantly lower amounts of intracellular GSH than tolerant individuals. In addition, the in vitro analysis of monocytes and platelets from sulfamethoxazole hypersensitivity patients challenged with sulfamethoxazole metabolites demonstrated an increased vulnerability of these cells. These pieces of evidence show a major role for oxidative stress in cytotoxicity of sulfamethoxazole and suggest a role of the redox status in the development of specific immunological drug reactions [16].

## 3. Genetics of Redox System and Their Relationship with Hypersensitivity Drug Reactions

### 3.1. Glutathione Transferases

GSTs are phase II multifunctional enzymes that play an important role in cellular protection by detoxifying numerous electrophilic compounds as well as regulating cellular redox homeostasis [50]. GSTs are categorized into cytosolic, mitochondrial, and microsomal families. There are seven classes within cytosolic GSTs (GSTA, GSTM, GSTP, GSTT, GSTO, GSTZ, and GSTS) that differ in their chemical, physical, and structural properties [50]. The effect of polymorphisms in the genes encoding these cytosolic enzymes, as well as that of gene duplications and deletions, has been extensively studied and their functional relevance in the context of drug detoxication has been highlighted [25,76,77,78,79]. Here, we will focus on their impact on DHRs development.

Glutathione S-transferase alpha (GSTA) class genes, comprised of 5 genes, is placed in a cluster on chromosome 6 [80]. Certain polymorphisms have been identified in the coding region of *GSTA1*, *GSTA2,* and *GSTA3*, although there is no clear evidence of their influence on the GSTAs activity [77]. Moreover, further studies have shown that GSTA1 haplotypes comprised of SNPs in the 5’ non-coding region may affect *GSTA1* expression levels [77]. Nevertheless, there are no reports of their impact on DHRs susceptibility.

The *GST Mu class* (*GSTM*) is located on chromosome 1. *GSTM* class are dimeric proteins with a tissue specificity expression [77]. The *GSTM1* locus is highly polymorphic, being the *GSTM1* null allele the most common allele in most populations studied so far. Thus, the homozygous deletion of the *GSTM1* gene leads to the absence of enzymatic activity [81]. Evidence of an association between *GSTM1* null genotypes and the development of skin eruptions caused by NSAIDs and β-lactam antibiotics has been reported [28], although this result was not confirmed in a recent study, possibly due to differences in the frequencies for the genetic variations between both cohorts [29]. Moreover, *GSTM1* null genotype has been related to the most severe and rare form of cutaneous drug reactions, Steven-Johnson syndrome, and toxic epidermal necrolysis (SJS/TEN) in HIV-positive patients receiving nevirapine [30]. Individuals carrying the *GSTM1* null genotype might show a low efficiency to detoxify the 12-sulfoxyl-nevirapine metabolite [30].

Drug-induced liver injury (DILI) is one of the most common DHRs and a recent updated meta-analysis has confirmed an association between *GSTM1* null genotypes and susceptibility to antituberculosis-DILI in South Asian individuals [31]. GSTM enzymes exert an essential role in detoxifying ROS and xenobiotics and individuals holding *GSTM1* null genotypes have shown an absence of GSTM activity [81]. Therefore, considering the interactions between the redox status and DHRs, it seems plausible that the absence of GSTM activity caused by *GSTM1* null genotypes may trigger an imbalance in redox status that may have an impact on the susceptibility to develop a DHR. Apart from the aforementioned *GSTM1* null genotype, the GSTM3*C haplotype has been functionally characterized, resulting in a gain of function, and several SNPs have been identified in the promoter region, but none have been shown to alter gene expression [77,82]. The effect of these SNPs in susceptibility to DHRs remains to be elucidated.

The *GST Pi class* (*GSTP*) is formed by a unique class gene, *GSTP1* which is placed on chromosome 11. Two nonsynonymous variants, rs1695 and rs1138272 have been extensively characterized and have shown differences in the GSTP1 activity in the metabolism of certain drugs [77,83]. The effect of the variant *GSTP1* rs1695 in DHRs is controversial. Thus, *GSTP1* rs1695 mutant homozygous genotype confers risk for skin drug eruption induced by NSAIDs, β-lactam antibiotics, and other drugs in a Jewish population [29] whereas this association was not found in a cohort of Turkish patients [28]. The *GSTP1* rs1695 causes the amino acid substitution Ile105Val, the mutant genotype has been associated with decreased activity in the GSH conjugation of the anticancer drug thiotepa [84]. Nevertheless, regarding NSAIDs and β lactams, this finding is still inconclusive, as there a lack of studies. In addition to these genetic associations, the characterization of GSTP1 oligomerization, which modulates its activity, by cyclopentanone prostaglandins and certain drugs exposes a direct crosstalk between inflammatory mediators and cellular redox homeostasis [85]. Therefore, these functional and genetic evidences support the need for additional studies in larger patient cohorts which might shed light on the role of this variant in DHRs.

The *GST* Theta class (*GSTT*) is comprised of two functional genes (*GSTT1* and *GSTT2*) which are placed on chromosome 22 [86]. Several SNPs in *GSTT1* have been functionally characterized. Thus, the mutant variants rs11550605, rs2266633, and rs2234953 might cause instability and low activity of GSTT1 [87,88]. The majority of *GSTT1* variants occur at a relatively rare frequency and are population-specific [89]. However, the gene deletion GSTT1*0 occurs frequently in most populations [77,89]. The GSTT1*0 variant has been associated with an increased risk of DHRs triggered by a variety of drugs, including NSAIDs and antibiotics in a cohort of Turkish patients [28]. Although this association was not confirmed in a Jewish population [29]. A certain number of studies have shown controversial results about the association between *GSTT1* null variant and DILI [31,33,34]. Nonetheless, meta-analysis studies did not confirm that association [31,90]. Lucena et al. demonstrated an association of *GSTM1-T1* double-null genotype and Spanish patients with co-amoxiclav hepatotoxicity [25]. Nevertheless, this finding was not observed in a larger cohort of European co-amoxiclav hepatotoxicity patients [32]. Therefore, the association found between *GST* variants and DILI patients seems to be more related to anti-tuberculosis drugs or NSAIDs than to co-amoxiclav.

The GSTT2 locus includes an inverted repeat of *GSTT2* named *GSTT2b* which is commonly deleted and occurs in linkage disequilibrium with the *GSTT1* null allele [91]. No association between *GSTT2* variants and susceptibility to DHRs has been described. In addition, there is a lack of evidence of an association between *GSTS, GSTZ,* or *GSTO* variants and the risk of developing DHRs [77].

### 3.2. Glutathione Peroxidases

GPX family is composed of eight isoenzymes in mammals. Their expression varies across different cells and tissues. These enzymes catalyze the reduction of hydrogen peroxide and lipid hydroperoxides using GSH as a reducing agent [48,92]. GPX1 is ubiquitous and its action contributes to diminish DNA damage and inhibit the synthesis of inflammatory mediators whereas GPX2 and GPX3 are overexpressed, mainly, in diverse cancer types [92,93].

The *GPX1* gene is located on chromosome 3p21.3 [93]. The non-synonymous *GPX1* rs1050450 (Pro200Leu) variant has been extensively studied and the mutant allele has been identified as a risk factor for lung cancer, cardiovascular or aging brain [94,95]. In addition, the *GPX1* rs1050450 mutant variant has been related to decreased GPX1 activity in cancer patients [96,97,98]. Concerning DHR, Lucena et al. described a significant association between the rs1050450 mutant homozygous genotype and the risk of developing cholestatic injury triggered by various drugs; including NSAIDs and β-lactam antibiotics [35]. This finding was not replicated in another cohort of European co-amoxiclav-related DILI patients [32]. Whether the impaired activity of *GPX1* rs1050450 mutant might enhance oxidative stress and consequently contribute to activate the inflammation pathway or generate drug reactive metabolites needs to be extensively studied.

### 3.3. Thioredoxin Reductases

Thioredoxin reductases (TrxR) are a family of NADPH-dependent enzymes that make part of the thioredoxin system. This system reduces ROS and represents the major H_2_O_2_ scavenger in mitochondria [99]. Three isoforms have been characterized in mammalians. TrxR1 is mainly located in the cytosol, while TrxR2 is in the mitochondria and TrxR 3 is predominantly expressed in maturing spermatids [100,101]. TrxR1 and TrxR2 are encoded by *TXNRD1* and *TXNRD2* genes which are placed on chromosomes 12 and 22; respectively [102].

A few studies have explored the role of *TXNRD1* genetic variants and the DHRs. These have been focused on DILI. Hence, Kwon et al. analyzed the SNPs *TXNRD1* rs10735393, rs4964287, and rs4595619, rs10861201, rs11111997, rs4246270, and rs4246271. The analysis of isolated SNPs did not reveal any association. However, an association was identified between the TTA haplotype, composed of *TXNRD1* rs10735393, rs4964287, and rs4595619, and DILI in a cohort of Korean patients [26]. In addition, the TCAGCC haplotype conformed by rs10735393, rs4964287, rs4595619, rs4246270, rs4246271, and rs11611385 was associated with hepatotoxicity induced by anti-tuberculosis drugs in Chinese female non-smokers [36]. However, these associations were not confirmed in a larger cohort of Chinese patients with DILI induced by anti-tuberculosis drugs [37]. The SNPs that conformed these targeted *TXNRD1* haplotypes are mainly located in intronic regions and there are not functional studies related to these SNPs. Therefore, studies aimed to determine the impact of these variants in the TXNRD1 capacity for reducing thioredoxins and their role in DHRs underlying mechanism are warranted.

### 3.4. Superoxide Dismutase

SODs are a family of enzymes responsible for the detoxication of superoxide anion radicals acting as an important defense mechanism against cellular damage [103]. Three forms have been described (SOD1, SOD2, and SOD3) and they are found in the cytoplasm, mitochondria, and extracellular space; respectively [103].

*SOD1* is localized on chromosome 21 and contains five exons. Several rare variants (rs202446, rs202447, rs4816405, and rs2070424) have been associated with an increased *SOD1* expression [104] although their functional significance is still unclear [105]. Among these variants, rs2070424 has been associated with DILI induced by anti-tuberculosis drugs in Korean patients [38]. Conversely, *SOD1* rs4816407 and rs1041740 were not associated in a Chinese cohort of patients with DILI induced by anti-tuberculosis drugs [39]. The essential SOD activity to protect against redox imbalance has been shown altered in patients with a non-immediate DHR [74]. This finding supports the link between the antioxidant scavenger enzymes and DHRs. Therefore, additional functional studies to determine whether this observed impaired activity is driven by SNPs in *SOD1* are required.

Concerning *SOD2,* which is located on chromosome 6, the nonsynonymous SNP rs4880 (Val16Ala) is the most studied. The mutant variant is associated with an augment of the SOD2 import into the mitochondrial matrix and reduced catalytic activity [106,107]. The *SOD2* homozygous genotype has been associated with the risk of developing DILI in Taiwanese and Spanish cohorts [35,40]. Both cohorts included a wide range of drugs inducing this pathology. Nevertheless, this association was not confirmed in Korean or Chinese patients with DILI induced by anti-tuberculosis drugs [37,38,39] nor European patients with co-amoxiclav DILI [32]. The discrepancies between these results might be explained due to ethnic differences in allele frequencies and the drugs involved in DILI development. These findings are keeping in line with the reduced catalytic activity associated with mutant homozygous *SOD2* rs4880. The impaired activity associated with this genotype is related to SOD2 transport efficiency into the mitochondria [106]. The mitochondria are the main source of ROS and SOD2 exerts a primordial role by reducing superoxide anion radical and protecting cells from redox imbalance [48]. Thereby, an altered SOD2 activity may evoke a redox imbalance which contributes to an alteration in inflammatory signaling pathways and drug reactive metabolites generation, and consequently the occurrence of DHRs.

The *SOD3* gene is placed on chromosome 4. The missense variants *SOD3* rs2536512 and rs1799895 and the following variants in non-coding region *SOD3* rs699473, rs2855262, and rs8192290, have been analyzed to investigate the possible association with DILI induced by anti-tuberculosis drugs. However, none were found to be associated with the risk [38,39]. Whether *SOD3* polymorphisms have a relevant role in the development of DHRs needs to be investigated more extensively.

### 3.5. Catalase

CAT plays an important role by discomposing H_2_O_2_, mainly at high H_2_O_2_ concentrations [108]. *CAT* has been localized on chromosome 11 and its genetic variations have been the subject of a previous review [109]. Hence, it has been reported that the *CAT* rs1001179 SNP, which is placed in the promoter region, may modulate the specificity of different transcriptional factor binding. In addition, individuals carrying the mutant T allele showed higher *CAT* expression levels in blood than homozygous for the C allele [110]. However, the results about the influence of this variant on CAT activity are controversial [109]. In addition, carriers of the variant *CAT* rs769214 have demonstrated a higher CAT activity in basal conditions [109,111]. Also, a certain number of *CAT* SNPs in the exon regions have been described [109]. A plethora of studies have associated *CAT* polymorphisms with a wide range of pathologies [109]. Nevertheless, there is a lack of studies involving *CAT* variants and DHRs.

### 3.6. Aldo-Keto Reductase

AKR protein superfamily is comprised of 15 different families which also contain multiple subfamilies [112]. AKRs catalyze the reduction of carbonyl groups to yield primary and secondary alcohols utilizing NADPH as a cofactor on a wide range of substrates [112]. AKR1 is the largest family and is subdivided into different subgroups. AKR1B1 catalyzes the conversion of glucose to sorbitol leading to redox imbalances and resulting in tissue injury which is linked to diabetic complications [113]. Moreover, AKR1B1 exerts a role in inflammatory processes. Hence, AKR1B1 exerts a role in inflammation modulation by synthesizing prostaglandin PGF2α which can regulate PGE_2_ production [59]. In turn, several prostaglandins such as PGA_1_ and PGE_2_ are capable to bind and inhibit AKR1B1 [114]. Furthermore, structural analysis of sulindac, a precursor of the NSAID sulindac sulfide, has revealed its capacity to inhibit AKR1B1 and AKR1B10 isoforms [115,116]. These interactions open new possibilities to explore the role of the AKR1B subfamily and the cellular redox system in the imbalance in prostaglandins and lipid mediators detected in NSAIDs hypersensitivity patients. *AKR1B1* is placed on chromosome 7 and it is highly polymorphic. Most of the association studies involving *AKR1B1* SNPs are related to the development of diabetes [117]. In addition, several polymorphisms have been associated with diverse pathologies [118]. The *AKR1B1* rs2229542 nonsynonymous variant has been associated with asthma and allergic rhinitis. It has been suggested that its occurrence might modify the AKR1B1 protein stability and consequently contribute to alterations in its function [119]. Moreover, the effect of *AKR1B1* rs5054, rs5056, rs5057, rs2229542, rs5061, and rs5062 variants on the activity over anticancer drugs doxorubicin and daunorubicin has been evaluated. However, none of these variants caused significant activity changes [120]. Concerning to AKR1C subfamily, its different isoforms metabolize multiple drugs such as NSAIDs, antipsychotics, and anticancer agents [121]. Interestingly, certain NSAIDs, such as flufenamic acid and indomethacin, have the ability to inhibit AKR1C3 activity [122]. In addition, acetylsalicylic acid and its metabolite salicylic acid were found to inhibit the isoform AKR1C1 activity [123]. Besides, different enzymes in the AKR1C subfamily may be involved in the nabumetone metabolism [124]. These findings reveal crosstalk between NSAIDs and redox status, which might contribute to the underlying mechanism of DHRs triggered by NSAIDs. *AKR1C1-4* genes are located on chromosome 10 [125]. Several polymorphisms have been described in these genes being associated with cancer or mental disorders [126,127,128] or with a decrease in enzyme activity [120]. However, there is a lack of data about the role of these variants on DHRs development.

### 3.7. Peroxiredoxin System

Peroxiredoxins (PRDXs) constitute a family of small non-seleno peroxidases. Six isoforms have been described in humans. They all catalyze peroxide reduction of H_2_O_2_, organic hydroperoxides, and peroxynitrite using the thioredoxin system as the electron donor [129,130]. There is scarce information on the involvement of *PRDXs* variants in the development of DHRs. Nevertheless, evidence for the contribution of several polymorphisms in these genes to drug metabolism variability and prognosis in breast cancer patients exist. Thus, the *PRDX4* rs518329 mutant homozygous genotype has been associated with decreased clearance of the anti-cancer chemotherapy drug docetaxel [131]. As the rs518329 is an intronic variant, the functional basis for the observed effect remains to be elucidated. Besides, the *PRDX6* rs4916362 which is located at a transcription factor binding site, and *PRDX6* rs7314 placed at a miRNA binding site, may modify the mortality risk in breast cancer patients [132]. Whether *PRDX* variants might exert a role in the susceptibility of DHRs need to be further studied.

## 4. Conclusions

Current evidence supports that GSTM1, GSTP1, GSTT1, TXNRD1, SOD1, and SOD2 enzymes may exert a role in the underlying mechanism of DHRs. Prominent findings include the association of *GSTM1* null or *SOD2* rs4880 and DHRs triggered by NSAIDs and β-lactam antibiotics [28,30,31,35,40], whereas other putative associations such as *GSTP1* rs1695, *GSTT1* null, *SOD1* rs2070424, or TXNRD1 haplotype require further studies [26,28,29,31,32,36,37,38].

Evidence indicates that, for reactions where the release of inflammatory mediators is not mediated by specific immunological mechanisms, redox status contributes to the initiation, the progress, and the resolution of inflammatory processes by interaction with COX enzymes, inflammatory mediators, and transcription factors. As for DHRs which imply the involvement of specific immunological mechanisms, the redox status contributes to the generation of reactive metabolites that might act like haptens and trigger the specific immune DHRs.

Also, the redox status of stressed cells might generate damage signals that would activate the antigen-presenting cells, thus resulting in increased expression of stimulatory molecules that trigger the specific immune response and contributing to the development of DHRs. A wide range of enzyme systems control the balance of the redox status, and the genes that encode these enzymes are polymorphic. Increasing evidence has demonstrated associations between polymorphisms in these genes and susceptibility to DHRs. Nevertheless, no large population studies have been carried out, and most studies published so far have been conducted in Asian patients only. This is a major limitation because gene variant frequencies for the above-mentioned genes greatly differ across human populations, and therefore, the association might not be extrapolated from one human population to another [133]. Furthermore, the association studies between genes involved in the redox status and NSAIDs hypersensitivity reactions are scarce and limited. Hence, in the light of the growing knowledge of the contribution of the redox system in the underlying mechanism of hypersensitivity drug reactions and the recent pharmacogenetic findings, the need for further research covering non-Asian populations, as well as a wide number of polymorphisms and DHRs, including different clinical entities, become evident.

## Figures and Tables

**Table 1:** Summary of variants analyzed in genes involved in redox status and the risk of drug hypersensitivity reactions development.

Gene	Variant	Functional Effect	Drug	Clinical Entity	Population	Number of Patients and Controls	MAF or Genotypic Frequencies %	P-Value; OR (95% CI)	Ref
*GSTM1*	*GSTM1* null	Absent GSTM1 activity	NSAIDs, β-lactam antibiotics, anticonvulsants	Maculopapular eruption, Erythema, Urticaria	Turkish	Patients: 36 Controls: 89	Patients: 0.5 Controls: 0.35	* <0.05; 2.27 (1.20–5.21)	[28]
*GSTM1*	*GSTM1* null	Absent GSTM1 activity	NSAIDs, β-lactam antibiotics, anticonvulsants, antidiuretic, statins, macrolide antibiotic	Urticaria, morbilliform rash, Steven-Johnson Syndrome, Photosensitive	Jewish	Patients: 50 Controls: 116	Patients: 0.40 Controls: 0.47	* 0.47; 1.35 (0.65–2.81)	[29]
*GSTM1*	*GSTM1* null	Absent GSTM1 activity	Nevirapine	Steven-Johnson Syndrome	African	Patients: 27 Controls: 78	Patients: 0.37 Controls: 0.16	* 0.027; 2.94 (1.10–7.85)	[30]
*GSTM1*	*GSTM1* null	Absent GSTM1 activity	Anti-tuberculosis drugs	DILI	South Asian	Patients: 311 Controls: 1200	Patients: 0.5 Controls: 0.43	** 0.005; 1.48 (1.12–1.95)	[31]
*GSTM1*	*GSTM1* null	Absent GSTM1 activity	Anti-tuberculosis drugs	DILI	East Asian	Patients: 408 Controls: 2324	Patients: 0.59 Controls: 0.55	** 0.12; 1.20 (0.95–1.52)	[31]
*GSTM1*	*GSTM1* null	Absent GSTM1 activity	Anti-tuberculosis drugs	DILI	South East Asian	Patients: 125 Controls: 60	Patients: 0.24 Controls: 0.17	** 0.26; 1.58 (0.71–3.49)	[31]
*GSTM1*	*GSTM1* null	Absent GSTM1 activity	Co-amoxiclav	DILI	European	Patients: 162 Controls: 326	Patients: 0.47 Controls: 0.56	* 0.08; 0.7 (0.5–1.0)	[32]
*GSTM1*	*GSTM1* null	Absent GSTM1 activity	Anti-infectives, NSAIDs, co-amoxiclav, cardiovascular therapy, others	DILI	Spanish	Patients: 154 Controls: 250	Patients: 0.558 Controls: 0.452	** 0.085; 1.53 (1.02–2.30)	[25]
*GSTM1*	*GSTM1* null	Absent GSTM1 activity	Anti-tuberculosis drugs	DILI	Indian	Patients: 51 Controls: 100	Patients: 0.49 Controls: 0.49	* 1.0; 1.0 (0.51–1.97)	[33]
*GSTM1*	*GSTM1* null	Absent GSTM1 activity	Anti-tuberculosis drugs	DILI	Indian	Patients: 50 Controls: 246	Patients: 0.42 Controls: 0.25	* 0.02; 2.14 (1.1–4.1)	[34]
*GSTP1*	rs1695 (Ile105Val)	Pharmacokinetic effect depends on substrate	NSAIDs, β-lactam antibiotics, anticonvulsants	Maculopapular eruption, Erythema, Urticaria	Turkish	Patients: 36 Controls: 89	Patients: 0.45 Controls: 0.40	* 0.56; n.a.	[28]
*GSTP1*	rs1695 (Ile105Val)	Pharmacokinetic effect depends on substrate	NSAIDs, β-lactam antibiotics, anticonvulsants, antidiuretic, statins, macrolide antibiotic	Urticaria, morbilliform rash, Steven-Johnson Syndrome	Jewish	Patients: 40 Controls: 120	Patients: 0.42 Controls: 0.29	* 0.038; 3.64 (1.08–12.28)	[29]
*GSTT1*	null	Absent GSTT1 activity	NSAIDs, β-lactam antibiotics, anticonvulsants	Maculopapular eruption, Erythema, Urticaria	Turkish	Patients: 36 Controls: 89	Patients: 0.31 Controls: 0.16	* <0.05; 2.48 (1.12–6.39)	[28]
*GSTT1*	null	Absent GSTT1 activity	NSAIDs, β-lactam antibiotics, anticonvulsants, antidiuretic, statins, macrolide antibiotic	Urticaria, morbilliform rash, Steven-Johnson Syndrome	Jewish	Patients: 50 Controls: 116	Patients: 0.40 Controls: 0.34	* 0.57; 0.76 (0.36–1.59)	[29]
*GSTT1*	*GSTT1* null	Absent GSTT1 activity	Anti-infectives, NSAIDs, co-amoxiclav, cardiovascular therapy, others	DILI	Spanish	Patients: 154 Controls: 250	Patients: 0.292 Controls: 0.232	** 0.394; 1.37 (0.87–2.15)	[25]
*GSTT1*	*GSTT1* null	Absent GSTT1 activity	Co-amoxiclav	DILI	European	Patients: 162 Controls: 326	Patients: 0.21 Controls: 0.20	* 0.81; 1.1 (0.7–1.7)	[32]
*GSTT1*	*GSTT1* null	Absent GSTT1 activity	Anti-tuberculosis drugs	DILI	Indian	Patients: 51 Controls: 100	Patients: 0.06 Controls: 0.03	* 0.41; 2.02 (0.39–10.39)	[33]
*GSTT1*	*GSTT1* null	Absent GSTT1 activity	Anti-tuberculosis drugs	DILI	Indian	Patients: 50 Controls: 246	Patients: 0.22 Controls: 0.12	* 0.08; 2.03 (0.9–4.4)	[34]
*GSTT1- GSTM1*	*GSTT1* null- *GSTM1* null	Absent GSTT1 and GSTM1 activities	Anti-infectives, NSAIDs, co-amoxiclav, cardiovascular therapy, others	DILI	Spanish	Patients: 154 Controls: 250	Patients: 0.182 Controls: 0.076	** 0.003; 2.70 (1.45–5.03)	[25]
*GSTT1- GSTM1*	*GSTT1* null- *GSTM1* null	Absent GSTT1 and GSTM1 activities	Co-amoxiclav	DILI	Spanish	Patients: 32 Controls: 250	Patients: 0.188 Controls: 0.076	** 0.037; 2.81 (1.06–7.46)	[25]
*GSTT1- GSTM1*	*GSTT1* null- *GSTM1* null	Absent GSTT1 and GSTM1 activities	Co-amoxiclav	DILI	European	Patients: 162 Controls: 326	Patients: 0.111 Controls: 0.123	* 0.77; 1.12 (0.6–2.1)	[32]
*GSTT1- GSTM1*	*GSTT1* null- *GSTM1* null	Absent GSTT1 and GSTM1 activities	NSAIDs	DILI	Spanish	Patients: 19 Controls: 250	Patients: 0.278 Controls: 0.076	* 0.001; 5.61 (1.99–16.0)	[25]
*GSTT1- GSTM1*	*GSTT1* null- *GSTM1* null	Absent GSTT1 and GSTM1 activities	Anti-tuberculosis drugs	DILI	Indian	Patients: 51 Controls: 100	Patients: 0.06 Controls: 0.11	* 0.39; 0.51 (0.13–1.90)	[33]
*GSTT1- GSTM1*	*GSTT1* null- *GSTM1* null	Absent GSTT1 and GSTM1 activities	Anti-tuberculosis drugs	DILI	Indian	Patients: 50 Controls: 246	Patients: 0.10 Controls: 0.02	* 0.007; 7.18 (1.7–32.6)	[34]
*GPX1*	rs1050450	The 200Leu variant is associated with lower GPX1 activity	Antiinfectives, antibacterials, Anti-tuberculosis drugs, NSAIDs, antiepileptics, others	DILI (Cholestatic)	Spanish	Patients: 50 Controls: 270	Patients: CC: 0.67 CT: 0.30 TT: 0.03 Controls: CC: 0.58 CT: 0.30 TT: 0.06	** 0.0112; 5.1 (1.6–16.0)	[35]
*GPX1*	rs1050450	The 200Leu variant is associated with lower GPX1 activity	Co-amoxiclav	DILI	European	Patients:157 Controls:334	Patients: CC: 47.8 CT: 43.3 TT: 8.9 Controls: CC: 41.0 CT: 5.4 TT: 53.6	* 0.25; 1.26 (0.9–1.9)	[32]
*TXNRD1*	rs10735393-rs4964287-rs4595619 TTA-haplotype	Unknown	Anti-tuberculosis drugs, antibiotics, antiepileptic drugs, NSAIDs	DILI	Korean	Patients: 118 Controls: 120	Patients: 0.309 Controls: 0.196	** 0.024; 1.79 (1.18–2.73)	[26]
*TXNRD1*	rs10735393, rs4964287, rs4595619, rs4246270, rs4246271, rs11611385 TCAGCC haplotype	Unknown	Anti-tuberculosis drugs	DILI	Chinese	Patients: 24 Controls: 223	Patients: 0.063 Controls: 0.012	* 0.036; 5.71 (0.92–35.56)	[36]
*TXNRD1*	rs11111997- rs10778322- rs4246270- rs4964782- rs4246271- rs11611385 CCAGGC haplotype	Unknown	Anti-tuberculosis drugs	DILI	Chinese	Patients: 461 Controls: 466	Patients: 0.186 Controls: 0.167	* 0.299; 1.135 (0.894–1.441)	[37]
*SOD1*	rs2070424 (A<G)	This rare variant is associated with an increased expression of SOD1 mRNA in the CEPH cell lines.	Anti-tuberculosis drugs	DILI	Korean	Patients: 84 Controls: 237	Patients: AA: 0.275 GA+GG:0.725 Controls: AA: 0.143 GA+GG:0.857	* 0.019; 2.26 (1.14–4.49)	[38]
*SOD1*	rs4816407	Unknown	Anti-tuberculosis drugs	DILI	Chinese	Patients: 116 Controls: 625	Patients: 0.453 Controls: 0.462	* 0.80; 0.96 (0.73–1.28)	[39]
*SOD1*	rs1041740	Unknown	Anti-tuberculosis drugs	DILI	Chinese	Patients: 118 Controls: 626	Patients: 0.360 Controls: 0.393	* 0.343; 0.87 (0.65–1.16)	[39]
*SOD2*	rs4880 (Val16Ala)	The Valine16 variant is associated with decreased mRNA stability	Anti-tuberculosis drugs, Antibiotics, NSAIDs, others	DILI	Taiwanese	Patients: 115 Controls: 115	Patients: TT: 0.557 TC+CC:0.443 Controls: TT: 0.757 TC+CC: 0.243	* 0.002; 2.44 (1.38–4.30)	[40]
*SOD2*	rs4880 (Val16Ala)	The Valine16 variant is associated with decreased mRNA stability	Antiinfectives, antibacterials, anti-tuberculosis drugs, NSAIDs, antiepileptics, others	DILI (Cholestatic/Mixed)	Spanish	Patients: 97 Controls: 270	Patients: TT: 0.20 TC: 0.44 CC: 0.36 Controls: TT: 0.31 TC: 0.49 CC: 0.20	** 0.0058; 2.3 (1.4–3.8)	[35]
*SOD2*	rs4880 (Val16Ala)	The Valine16 variant is associated with decreased mRNA stability	Antiinfectives, antibacterials, anti-tuberculosis drugs, NSAIDs, antiepileptics, others	DILI	Spanish	Patients: 180 Controls: 270	Patients: TT: 0.21 TC: 0.49 CC: 0.30 Controls: TT: 0.31 TC: 0.49 CC: 0.20	** 0.02; 1.7 (1.1–2.6)	[35]
*SOD2*	rs4880 (Val16Ala)	The Valine16 variant is associated with decreased mRNA stability	Anti-tuberculosis drugs	DILI	Chinese	Patients: 461 Controls: 466	Patients: TT: 0.725 TC: 0.245 CC: 0.026 Controls: TT: 0.732 TC: 0.245 CC: 0.027	* 0.80; 1.11 (0.485–2.559)	[37]
*SOD2*	rs4880 (Val16Ala)	The Valine16 variant is associated with decreased mRNA stability	Anti-tuberculosis drugs	DILI	Korean	Patients: 84 Controls: 237	Patients: TT: 0.747 CT+CC: 0.253 Controls: TT: 0.789 CT+CC: 0.211	* 0.399; 1.29 (0.71–2.35)	[38]
*SOD2*	rs4880 (Val16Ala)	The Valine16 variant is associated with decreased mRNA stability	Anti-tuberculosis drugs	DILI	Chinese	Patients: 117 Controls: 626	Patients: 0.175 Controls: 0.122	** 0.190; 1.53 (1.05–2.23)	[39]
*SOD2*	rs4880 (Val16Ala)	The Valine16 variant is associated with decreased mRNA stability	Co-amoxiclav	DILI	European	Patients: 158 Controls: 331	Patients: TT: 0.258 TC: 0.503 CC: 0.239 Controls: TT: 0.257 TC: 0.48 CC: 0.263	* 1.0; 1.02 (0.7–1.6)	[32]
*SOD3*	rs1799895	Unknown	Anti-tuberculosis drugs	DILI	Korean	Patients: 84 Controls: 237	Patients: GG: 0.458 GA+AA: 0.542 Controls: GG: n.a. GA+AA: n.a.	* 0.962; 0.99 (0.59–1.65)	[38]
*SOD3*	rs2536512	Unknown	Anti-tuberculosis drugs	DILI	Korean	Patients: 84 Controls: 237	Patients: CC: 0.905 CG+GG: 0.095 Controls: CC: 0.945 CG+GG: 0.055	* 0.191; 1.86 (0.73–4.72)	[38]
*SOD3*	rs699473 C>T	The C variant is more effective at competing DNA-protein binding	Anti-tuberculosis drugs	DILI	Chinese	Patients: 118 Controls: 626	Patients: 0.352 Controls: 0.341	* 0.629; 1.08 (0.80–1.44)	[39]
*SOD3*	rs2536512	Unknown	Anti-tuberculosis drugs	DILI	Chinese	Patients: 118 Controls: 624	Patients: 0.352 Controls: 0.342	* 0.777; 1.04 (0.78–1.40)	[39]
*SOD3*	rs2855262	Unknown	Anti-tuberculosis drugs	DILI	Chinese	Patients: 118 Controls: 626	Patients: 0.369 Controls: 0.362	* 0.842; 1.03 (0.77–1.38)	[39]
*SOD3*	rs8192290	Unknown	Anti-tuberculosis drugs	DILI	Chinese	Patients: 118 Controls: 628	Patients: 0.064 Controls: 0.094	* 0.366; 1.31 (0.73–2.34)	[39]

* *p*-value; ** adjusted *p*-value for multiple comparisons.
